# Traditional Medicine in China, Korea, and Japan: A Brief Introduction and Comparison

**DOI:** 10.1155/2012/429103

**Published:** 2012-10-24

**Authors:** Hye-Lim Park, Hun-Soo Lee, Byung-Cheul Shin, Jian-Ping Liu, Qinghua Shang, Hitoshi Yamashita, Byungmook Lim

**Affiliations:** ^1^School of Korean Medicine, Pusan National University, Yangsan 626-870, Republic of Korea; ^2^Centre for Evidence-Based Chinese Medicine, Beijing University of Chinese Medicine, Beijing 100-029, China; ^3^Graduate School of Health Sciences, Morinomiya University of Medical Sciences, Osaka 559-0034, Japan

## Abstract

*Background and Purpose*. Traditional medicine (TM) has been widely used in China (including the Taiwan region), Korea, and Japan. The purposes of this paper are to summarize the basic data on TM systems in these three countries and to compare them in terms of overall policy, education, and insurance. *Methods*. Government websites, national statistics, and authoritative papers from each country were fully searched. Further data were gathered by TM experts from each country. *Results*. China and Korea showed similar patterns in TM systems, whereas Japan showed different patterns. In China and Korea, TM was practiced in a dual system with conventional medicine (CM), and TM education was 6-year training programs on average for TM doctors, and acupuncture, moxibustion, and cupping were completely insured. Whereas, CM was dominant in Japan, and TM was practiced by each health care worker who has received different TM education respectively, and main TM therapies were partially insured. *Conclusions*. TM was developed similarly or somewhat differently based on differences in cultural background and national policies in East Asia. We cautiously propose that this study could contribute to the development of TM and also be used for reference in complementary and alternative medicine systems.

## 1. Introduction 

Traditional medicine (TM) is defined as indigenous medicine that is used to maintain health and to prevent, diagnose, and treat physical and mental illnesses differently from allopathic medicine based on theories, beliefs, and experiences [1]. In East Asian countries—especially China (including the Taiwan region), Korea, and Japan—the main therapeutic methods of TM consist of acupuncture, moxibustion, cupping, herbal medicines (called Kampo in Japan), and manual therapies (called Tuina in China, Chuna in Korea, and Anma-massage-Shiatsu and Judo therapy in Japan) [2]. 

East Asian medicine is known to have originated in China about 3,000 years ago [3]. It was introduced to Korea with Buddhism and to Japan with Chinese culture beginning in the 6th century [4, 5]. It has been widely used following a long history, and practices in the three countries strongly influenced each other. Before Western culture was introduced to East Asia in 19th century, TM was the main medical system used to treat all types of diseases [2]. 

Each country has developed unique TM systems [6] with its own name: traditional Chinese medicine (TCM) for China, traditional Korean medicine (TKM) for Korea, and Oriental medicine for Japan. For example, Sasang Constitutional medicine of TKM and Kampo medicine of Japan have different characteristics from TCM [3, 7]. Such differences might result from their unique national histories, cultural differences, different acceptance of Western culture, and influences of the Second World War [8]. In the following, we write “TM” when we are not differentiating practices by country.

TCM accounts for around 40% of all health care delivered, and it is used to treat approximately 200 million patients annually in China [9]. Also 69% of the Korean population has experienced TKM [10], and 60–70% of allopathic doctors in Japan prescribe herbal medicines for their patients [9]. TM was included in the national health care systems of the three countries and developed in national policies based on historical and cultural background that differs from that of complementary and alternative medicine (CAM) in Western society [11]. 

Although data on TM in the three countries are available from the World Health Organization Western Pacific Region (WHO WPRO), it was difficult to find comparable data for concrete analyses. Information on TM systems in these three countries has not been well reported to Western countries, so it is meaningful to introduce that information to Western society by comparing practices in the three countries.

In this paper, China (including the Taiwan region), Korea, and Japan were selected because these countries are geographically close together and have historically had stronger influences on each other than other East Asian countries and because TM has played an important role in their medical service systems [8]. We gathered information and data on each country's current status of national policies and resources, formal education system, and medical insurance coverage of TM. These data will be especially useful to illustrate differences from CAM.

This paper seeks to understand the practices of each country by analyzing the basic data mentioned above. A further aim of this study is to define a future strategy to contribute to the development of TM systems in the three countries based on the results and to provide a reference or base document that can be used in CAM systems in Western countries.

## 2. Methods

### 2.1. Data Sources

We mainly searched official government websites, national statistics, and authoritative papers from databases of each country to obtain publicly available information about national policies and resources, formal education, and medical insurance coverage of TM. Additionally, the websites of WHO WPRO and international organizations related to TM were carefully searched. When desired information could not be located, we tried to collect information by contacting related associations, educational institutions, and governmental agencies by phone or e-mail. 

#### 2.1.1. Data for National Policies

Our main data sources were official government health websites: Mainland China (Ministry of Health, http://www.moh.gov.cn), the Taiwan region (Department of Health, http://www.doh.gov.tw), Korea (Ministry of Health and Welfare, http://www.mw.go.kr), and Japan (Ministry of Health, Labour, and Welfare, http://www.mhlw.go.jp). 

#### 2.1.2. Data for Medical Resources/Facilities

Data were collected from recent statistical yearbooks issued in each country, websites providing national statistics for mainland China (State Administration of TCM, http://www.satcm.gov.cn) and the Taiwan region (Directorate-General of Budget, Accounting, and Statistics, http://eng.dgbas.gov.tw/mp.asp?mp=2), official government websites of health for Korea and Japan, and an additional website about Kampo doctor (the Japan Society for Oriental Medicine, http://www.jsom.or.jp).

#### 2.1.3. Data on the Formal Education System

Data were collected and analyzed from all relevant websites of universities offering education in TM in mainland China, the Taiwan region, Korea, and Japan and from the 2009 Yearbook of TKM. The website of the Education Ministry of each country was also searched for relevant information. 

#### 2.1.4. Data on Medical Insurance and Herbal Drug Monitoring Systems

The data sources were the official government websites mentioned above. Websites of the WHO (World Health Organization, http://www.who.int), FHH (Forum for the Harmonization of Herbal Medicines, http://www.fhhm.net), KFDA (Korea Food and Drug Administration, http://www.kfda.go.kr/eng/index.do), PVNet of Korea (Pharmacovigilance Research Network, http://www.pvnet.or.kr), and NIHS of Japan (National Institute of Health Sciences, http://www.nihs.go.jp) were searched.

#### 2.1.5. General Information

To gather as much further information as possible, we searched well-known databases such as Google and PubMed using sensitive search terms. Additionally, four Korean medical databases (DBPIA, National Assembly Library, RISS, and Korean Traditional Knowledge Portal) and a Chinese medical database (China National Knowledge Infrastructure, http://www.cnki.net) were searched in more depth. 

### 2.2. Summary and Analysis of Data

The primary authors (HLP and BCS) predefined the three major criteria of the health service system as national policies and resources, formal education system, and medical insurance. Related information from each country was gathered according to the criteria, and we attempted to find all necessary data by searching the databases mentioned above. The accuracy of all collected data was checked, and then the information was confirmed by coauthors of each country. All coauthors of each country participated actively in this research as experts on TM systems. Remained missing (or unsearched) data for each country were collected from coauthors through their own documents, additional databases of each author, or personal contact. Quantitative data between countries were calculated exactly or estimated. Disagreements between authors were resolved by e-mail discussion. Finally, the data for each country were summarized, analyzed, and compared, and recommendations for future strategy were developed. 

## 3. Results and Discussion

### 3.1. Brief Historical Differences of the Development of TM Systems

**Figure 1:**
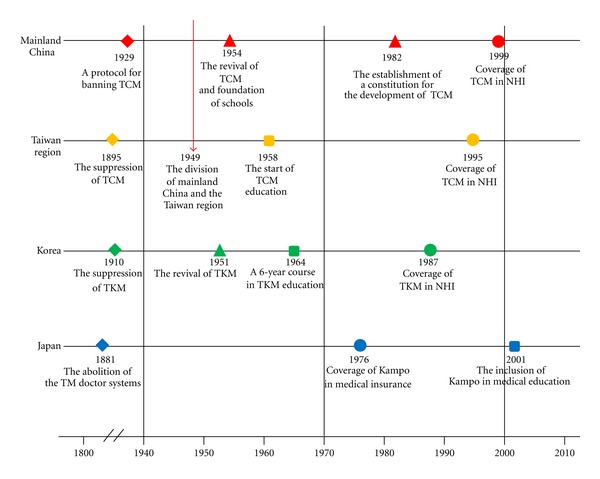
Main historical events related to traditional medicine in China (including the Taiwan region), Korea, and Japan. NHI: National Health Insurance; TCM: traditional Chinese medicine; TKM: traditional Korean medicine.

Brief historical differences of the development of TM systems in each country were summarized in Figure 1 to establish a more in-depth understanding of the three parts.

### 3.2. National Policies and Resources of TM

National policies and medical resources/facilities of TM in China, Korea, and Japan are listed in Table 1. All main therapeutic methods of TM were included in the national health care system in mainland China, the Taiwan region, and Korea, whereas Japan included only most Kampo (galenicals or herbal mixtures) and some acupuncture.

TM doctors, whose activities are defined by law and regulated by the government of each country, have three different specialties in mainland China: TCM, integrative medicine, and other ethnic medicine doctors. Only one kind of doctor, TCM (or TKM) doctors, are found in the Taiwan region and Korea, respectively, in contrast with conventional medicine (CM) doctors (including dental doctors). The percentage of TM doctors in medical facilities (hospitals and clinics) was the highest in Korea, at 15.26%, followed by mainland China, at 12.63%, and the Taiwan region, at 9.69%. For Japan, the percentage of Kampo doctors who are medical doctors and members of a nongovernmental academic society, the Japan Society for Oriental Medicine, was estimated to be 0.65% in 2008.

The ratio of medical facilities practicing CM to facilities practicing TM was 24.99 : 1 (CM : TM) for mainland China (including TCM, integrative medicine, and ethnic medicine), 3.36 : 1 for the Taiwan region, and 2.50 : 1 for Korea. In Japan, hospitals and clinics are regarded as CM facilities even if TM is practiced there, so the ratio for traditional Japanese medicine was recorded as zero. 

The Ministry of Health and Welfare of Korea implemented a TKM specialist training system in 1999 that follows the model of the CM specialist system. Medical care is provided in 8 departments: internal medicine, gynecology, pediatrics, neuropsychiatry, acupuncture and moxibustion, ophthalmology-otorhinolaryngology-dermatology, rehabilitation medicine, and Sasang Constitutional medicine. After obtaining a TKM license, a practitioner can become a TKM specialist through 4 years of additional training in a designated TKM hospital (a 1-year internship and a 3-year resident course). These practitioners accounted for 9.1% of all TKM licensed doctors by 2009 [12]. 

In Japan, CM doctors can practice any style of TM including acupuncture and Kampo in addition to CM, but not every doctor practices TM. Some of them can become Kampo doctors (0.65% in 2008) by joining the Japan Society for Oriental Medicine and studying Kampo [13] and prescribe Kampo using concentrated herbal extracts to patients. Acupuncture-moxibustion therapists can provide acupuncture and moxibustion in their medical service, Anma-massage-Shiatsu therapists provide Anma and Shiatsu, and Judo therapists (who were called “bonesetter” in the past) practice Judo therapy.

### 3.3. Formal TM Education System

The formal education system of TM in the three countries is summarized in Table 2. The ratios of the numbers of schools teaching CM and TM in each country were 4.59 : 1 (CM : TM) for mainland China, 3.67 : 1 for the Taiwan region, and 3.42 : 1 for Korea. 

Japan had 80 universities teaching CM for medical doctors; 11 universities, 88 professional schools, and approximately 70 schools for the blind teaching acupuncture, moxibustion, or Anma-massage-Shiatsu; and 12 universities and 92 professional schools for Judo therapists as of the year 2012. 

The TM education system of mainland China mostly consists of a 5-year course culminating in a bachelor's degree, accounting for more than 90% of graduates, and a 7-year course offering a bachelor's degree and a master's degree. Students who complete the 5-year course should complete an additional 1 year of clinical training to obtain a national license, and students enrolled in the 7-year course should write a master's thesis to earn a master's degree [14]. Additionally, there are a few other type of TM education, such as a 6-year bachelor's degree program (4.5 years of basic medicine and clinical theory and 1.5 years of clinical training) and a new 8-year course in which students earn bachelor's, master's, and doctoral degrees. The TM education system of the Taiwan region consists of two educational pathways: an 8-year course and a 5-year postbaccalaureate program. After graduation, only students who complete the 8-year course are eligible to obtain two kinds of licenses, Doctor of Chinese Medicine and Doctor of Medicine, after 1 year of clinical training in TCM and another year of training in CM. The TM education system of Korea consists of two educational pathways with a specialization in TKM: a 6-year program offered by 11 private universities is the main type, accounting for 92% of graduates, and a 4-year postbaccalaureate program is offered by 1 national university (8%). Students in the Taiwan region and Korea can enroll in their respective postbaccalaureate programs after finishing a 4-year bachelor's degree in another specialization and should write a master's thesis to earn a master's degree. Japan has a separate education system for each type of TM therapy. Medical universities of Japan teach mainly CM and have begun teaching TM (focusing mainly on Kampo, not acupuncture) more than 8 times (1 time for only 90 minutes) since 2001 [15]. The Kampo training is informal, and additional education from the Japan Society for Oriental Medicine is required after graduating from a medical university. The education system for acupuncturists, moxibustion therapists, Anma-massage-Shiatsu therapists, and Judo therapists mainly consists of two educational pathways: a 4-year university program and a 3-year course offered by professional schools and schools for the blind.

### 3.4. Medical Insurance Coverage of TM

All three countries have insured TM procedures: mainland China since 1951; the Taiwan region since 1995; Korea since 1987; and Japan since 1976, only for Kampo (Table 3). Mainland China had the most reimbursable treatments: acupuncture, moxibustion, cupping, and manual therapies are completely covered, and herbal medicines are partially covered. Acupuncture, moxibustion, and cupping were usually completely covered by medical insurance in mainland China, the Taiwan region, and Korea. Herbal medicines are divided roughly into two types: concentrated herbal extracts provided by a drug company according to GMP and decoctions of raw herbs prepared in medical facilities. In mainland China, herbal extracts are insured if they are accorded to the nominated indication (nominated to be applicable for specific diseases), and most of decoctions of raw herbs are also insured except for very precious medicine, herbal paste, and medicine without the nominated indication; herbal extracts are mostly insured, but decoctions of raw herbs are not in Korea; meanwhile, because only herbal extracts based on GMP are used in Japan, they are completely covered. Coverage of manual therapies was different in each country. Data on the proportion of reimbursement for TM treatments were not available for mainland China and Japan, whereas the Taiwan region showed a reimbursement rate of 5.59%, and Korea 4.04%. 

### 3.5. Herbal Drug Monitoring Systems

Adverse drug reactions (ADRs), including to herbal drugs, were tracked in mainland China, Japan, and Korea, which are members of the WHO Pharmacovigilance Programme [16] and the Forum for the Harmonization of Herbal Medicines [17]. In particular, Japan's Pharmaceuticals and Medical Devices Agency included a system for compensating for ADRs [10]. The ADRs reporting system of the Taiwan region was not connected with the WHO Pharmacovigilance Programme [16], but a separate monitoring system for herbal drugs was operated with Western drugs only in the Taiwan region. Mainland China reported the most ADRs to herbal drugs, at 14-15%, followed by Japan and Korea at 2.23% and 2-3% (estimated data), respectively [10] (Table 3). 

### 3.6. Similarities and Differences in TM Systems

China (including the Taiwan region) and Korea had similar patterns of national policies and resources, formal education system, and medical insurance coverage of TM, which is offered in parallel with CM for treating illness. In those countries, TM doctors is defined by law and regulated by the government. TM practitioners accounted for 9.69% to 15.26% of all doctors, and the number of medical facilities (hospitals and clinics) practicing TM was 3.85–28.6% of the number of facilities offering CM. The TM education system in these countries was a 6-year course (range 5–8) including more than 1 year of clinical training on average in TM practice, and education of each therapy of TM is connected to the others based on TCM (or TKM) theory. Students obtain a bachelor or master's degree after graduation and become eligible to apply for the national license examination offered by the government. The main therapeutic methods of TM were covered by national health insurance completely or partially. 

TM has taken a secondary role to CM in Japan, which showed different patterns for the three aspects of TM compared to the other countries. In Japan, each therapeutic method of TM can be practiced by CM doctors, acupuncturists, moxibustion therapists, Anma-massage-Shiatsu therapists, and Judo therapists separately and is taught individually. Most therapies of TM were covered partially by medical insurance, but herbal extracts provided according to GMP were completely covered.

### 3.7. Future Strategy

Based on the information provided, we conclude that it would be meaningful to establish minimal standards for the situation of each country and to develop a plan to improve patients' health and safety. In Korea, for example, as in other countries, the TM curriculum consisted of 60% CM as biomedicine and 40% TM [12] on average, and the aim of the program is to cultivate doctors who can diagnose disease and treat patients with their knowledge of both CM and TM. Therefore, we suggest that a minimum 5-year (average 6 years; 6416 hours) course including 1 year of clinical training would be suitable for TM education. Additionally, acupuncture, moxibustion, cupping, manual therapies, and herbal medicines were covered by public medical insurance in three countries, and research has gradually yielded evidence of their efficacy [18–20]. However, the evidence is still not significantly convincing, so further research should be performed to establish the efficacy of each therapy for more diseases. Because safety of herbal medicines has recently become an important issue [21], management or control of these substances is very important. Therefore, active reporting and systematic management of ADRs in connection with the WHO Pharmacovigilance Programme would be needed to establish an herbal drug monitoring system. The safety of herbal drugs has a significant influence on patients' health, so separate monitoring systems for herbal drugs and Western drugs, as used in the Taiwan region, may be a useful way to track ADRs caused by herbal medicines.

### 3.8. Differences between TM and CAM

We discovered substantial differences between the TM practices of three East Asian countries, especially TCM (or TKM), and CAM in Western society. First, TCM (or TKM) has been considered as a dual medical system with CM, unlike CAM. Second, the practice of and training in each therapeutic method of TM was combined under TCM (or TKM) theory, whereas in Western society, each therapy is offered individually, either together with or in place of CM [22]. The use of acupuncture has become common in Western society, as there are an estimated 15,000 acupuncturists in Europe [9], but the situation is different for other therapies. We think this difference arises from differences in cultural background. Third, the main therapies of TM were covered, completely or partially, by national health insurance, unlike in Western society, where only a few therapies of TM are covered by private health insurance. Eventually, TM infrastructure, from national licenses and resources, education, and medical insurance coverage to monitoring of herbal medicines, was developed organically, and the TM systems in each of the three countries influenced the others.

### 3.9. Values and Limitations

Our research has some limitations that should be mentioned. First, some data were limited or deficient due to their limited sources, and the information may be biased despite our efforts to obtain objective data. Additionally, the abilities to obtain mean data and propose future strategy were limited because each country had different policies, education, and medical insurance coverage related to TM. 

Nevertheless, this paper is valuable that TM systems in three East Asian countries were examined in the three aspects for the first time. Basic data of each country were compared as a basis for future strategy. Furthermore, the information presented here could be used for reference in research on CAM in Western society.

## 4. Conclusions

TM was developed similarly in China and Korea and somewhat differently in Japan. Additionally, substantial differences were discovered between the TM practices of the three East Asian countries and CAM in Western society in the three aspects. We think this difference arises from differences in cultural background and national policies. 

We cautiously propose that this study not only describes TM in the three countries but also contributes to providing improved medical services for TM to patients. Furthermore, the findings can be used as a basis for policy making, education, and insurance coverage decisions concerning CAM in Western society.

## Figures and Tables

**Table 1:** National policies and medical resources/facilities related to traditional medicine in China (including the Taiwan region), Korea, and Japan.

Country (year of most recent data)	Mainland China (2010)				Taiwan region (2009)		Korea (2009)		Japan (2009)	
Main therapeutic methods of TM included in national health care system	All				All		All		Some^a^	
Name	TCM				TCM		TKM		OM or TJM (recently proposed)	
Administration structure	MOH				DOH		MOHW		MHLW	

Legal definition of doctors	CM				CM		CM		CM	
	TCM				TCM		TKM			
	IM									

Percentage of TM doctors in medical facilities (%, TM doctors/total doctors∗100)	Ethnic medicine									
	TM doctors^b^ 12.63 (TCM doctors 10.19 IM doctors 1.22 other ethnic medicine doctors 1.22)				TCM doctors^b^ 9.69		TKM doctors^b^ 15.26 (9.1% of TKM doctors are specialist)		Kampo doctors^c^ 0.65 (2008)	

	CM	TCM	IM	Ethnic M	CM	TCM	CM	TKM	CM	TJM

Hospitals (beds)	16,794	2,778	256	198	496	18	2,337	151	8,739	0^d^
	(2,670,680)	(424,244)	(35,234)	(11,811)	(134,489)	(227)	(396,495)	(8,694)	(1,601,476)	(0)
Clinics (beds)	92,453 (7,981)	937 (596)	192 (185)	11 (4)	10,361 (20,381)	3,217 (1,524)	27,289 (91,762)	11,705 (944)	99,635 (141,817)	0^d^ (0)

Total (beds)	109,247 (2,678,661)	3715 (424840)	448 (35419)	209 (11815)	10,857 (154,870)	3,235 (1,751)	29,626 (488,257)	11,856 (9,638)	108,374 (1,743,293)	0^d^ (0)

CM: conventional medicine; DOH: Department of Health; IM: integrative medicine; MHLW: Ministry of Health, Labour, and Welfare; MOH: Ministry of Health; MOHW: Ministry of Health and Welfare; OM: oriental medicine; TCM: traditional Chinese medicine; TJM: traditional Japanese medicine; TKM: traditional Korean medicine; TM: traditional medicine.

^a^In Japan, most Kampo (galenicals or herbal mixtures) and some acupuncture are included in the national health care system.

^b^In China (including the Taiwan region) and Korea, TM doctors can practice all main therapeutic methods of TM, including acupuncture, moxibustion, cupping, herbal medicines, and manual therapies.

^c^In Japan, CM doctors can practice any style of TM including acupuncture and Kampo. Acupuncture is mainly practiced by acupuncturists, moxibustion by moxibustion therapists, Anma and Shiatsu by Anma-massage-Shiatsu therapists, and Judo therapy by Judo therapists. Some CM doctors are Kampo doctors belonging to the Japan Society for Oriental Medicine.

^d^In Japan, all medical facilities called a “hospital” or “clinic” in Japanese are considered as CM facilities, even if TM is partially practiced there. Acupuncture clinics (“Shinkyu-In” in Japanese) are not regarded as clinics in Japan.

**Table 2:** Formal education system of traditional medicine in China (including the Taiwan region), Korea, and Japan.

Country	Are there schools teaching TM?	Program duration	Composition	Degree	Specialization	License
Mainland China	Yes	5-year course	Basic medicine + clinical theory (3.5 years) clinical training (1.5 years)^a^	Bachelor's	TCM IM	DCM DIM
						
		7-year course	Basic medicine + clinical theory (5 years) clinical training (1.5 years) thesis (0.5 years)	Bachelor's and master's	TCM IM	DCM DIM

Taiwan region	Yes	8-year course	Basic medicine + clinical theory (6 years) clinical training (2 years)^b^	Bachelor's	TCM and CM	DCM and MD
						
		Postbaccalaureate program (5 years)^c^	Basic medicine + clinical theory (4 years) clinical training (1 year)	Master's	TCM	DCM

Korea	Yes	6-year course	Basic medicine + clinical theory (5 years) clinical training (1 year)	Bachelor's	TKM	DKM
						
		Postbaccalaureate program (4 years)^c^	Basic medicine + clinical theory (3 years) clinical training (1 year)	Master's	TKM	DKM

Japan	No (for MD)	—	—	Bachelor's	CM^d^	MD^d^
						
	Yes (for acupuncturists, moxibustion therapists, Anma-massage-Shiatsu therapists, and Judo therapists)	University (4 years)	Basic medicine + clinical theory clinical training (years not specifiable)	Bachelor's	Acupuncture, moxibustion, and Judo therapy (Anma-massage-Shiatsu in one university)	Licensed acupuncturist, moxibustion therapist, Anma-massage-Shiatsu therapist, and/or Judo therapist
						
		Professional school or school for the blind (3 years)	Basic medicine + clinical theory clinical training (years not specifiable)		Acupuncture, moxibustion, Anma-massage-Shiatsu, and Judo therapy	Licensed acupuncturist, moxibustion therapist, Anma-massage-Shiatsu therapist, and/or Judo therapist

CM: conventional medicine; D: doctor of; IM: integrative medicine; MD: doctor of medicine; OM: oriental medicine; TCM: traditional Chinese medicine; TKM: traditional Korean medicine; TM: traditional medicine

^a^In the 5-year course offered in China, students should complete an additional 1 year of clinical training to obtain a national license.

^b^Clinical training in the 8-year course offered in the Taiwan region consists of 1 year of training in TCM and another year of training in CM.

^c^Students in the Taiwan region and Korea can enter a postbaccalaureate program after finishing a 4-year Bachelor's Degree program in another specialization and should write a Master's thesis to earn a Master's Degree.

^d^Partial education about Kampo is included in CM education.

**Table 3:** Medical insurance and herbal drug monitoring systems of traditional medicine in China (including the Taiwan region), Korea, and Japan.

Country	Mainland China	Taiwan region	Korea	Japan
*Medical insurance *				
Insurance system	NHI and commercial insurance	NHI	NHI	8 kinds of health insurance
Type	Public (NHI) and Private (commercial insurance)	Public	Public	Public
Year TM implemented	1951^a^	1995	1987	1976 (for Kampo)
Reimbursable items				
Acupuncture	*⬤*	*⬤*	*⬤*	▲
Moxibustion	*⬤*	*⬤*	*⬤*	▲
Cupping	*⬤*	Data not available	*⬤*	X
Herbal medicines (Kampo in Japan)	▲	▲	▲	*⬤*
Manual therapies	*⬤*	*⬤*	▲^b^	▲^c^
Proportion (%, TM reimbursement/total reimbursement*100)	Data not available	5.59 (2009)	4.04 (2009)	Data not available

*Herbal drug monitoring systems *				
Administration structure	MOH and SFDA	CCMP of DOH	KFDA	MHLW and PMDA
Is the country a member of the WHO Programme?	Yes	No	Yes	Yes
Are herbal and Western drugs monitored separately?	No	Yes	No	No
No. of ADR reports for herbal drugs (%, TM reporting/total reporting*100)	14~15	Data not available^d^	2~3^d^	2.23

ADR: adverse drug reaction; CCMP: Committee on Chinese Medicine and Pharmacy; DOH: Department of Health; KFDA: Korea Food and Drug Administration; MHLW: Ministry of Health, Labor and Welfare; MOH: Ministry of Health; NHI: National Health Insurance; PMDA: Pharmaceuticals and Medical Devices Agency; SFDA: State Food and Drug Administration; TM: Traditional Medicine

*⬤*: Completely covered, ▲: partially covered, X: not covered.

^a^Medical insurance coverage of TCM was implemented in all insurance systems from the beginning of new China in 1951. In 1999, the new NHI system was established, and TCM remained an important element.

^b^In Korea, manual therapies (Chuna) are covered by car insurance in the event of an accident, not by NHI.

^c^In Japan, massage therapy is covered when performed in hospital.

^d^In total, 507 ADRs to herbal drugs were reported from August 2001 to November 2005, but the relevant data could not be obtained for the Taiwan region, and an estimated number was presented in Korea.

## References

[1] World Health Organization Media Centre Traditional medicine. http://www.who.int/mediacentre/factsheets/fs134/en.

[2] Cheung F (2011). TCM: made in China. *Nature*.

[3] Yu F, Takahashi T, Moriya J (2006). Traditional Chinese medicine and kampo: a review from the distant past for the future. *Journal of International Medical Research*.

[4] Kobayashi A, Uefuji M, Yasumo W (2010). History and progress of Japanese acupuncture. *Evidence-Based Complementary and Alternative Medicine*.

[5] Li CM Books originated from China and their effect to ancient Korea.

[6] Cha WS, Oh JH, Park HJ, Ahn SW, Hong SY, Kim NI (2007). Historical difference between traditional Korean medicine and traditional Chinese medicine. *Neurological Research*.

[7] Kim JY, Pham DD, Koh BH (2011). Comparison of sasang constitutional medicine, traditional chinese medicine and ayurveda. *Evidence-Based Complementary and Alternative Medicine*.

[8] Sung HJ, Shin HK (1997). *Comparison and Research on Policy of Traditional Medicine in Korea and Three Countries of East Asia*.

[9] World Health Organization (2002). *WHO Traditional Medicine Strategy 2002–2005*.

[10] Lee SD (2007). *The Study for Activation and Development of Herbal Adverse Reaction Reporting System*.

[11] Ryu GC, Lee HW, Oh SJ, Park CJ (2005). International competitiveness and tasks of Korean traditional medicine from the perspective of international comparison of curricula and research. *Journal of Korean Institute For Health and Social Affairs*.

[12] (2010). *Korean Medicine Yearbook 2009*.

[13] Korean Institute of Oriental Medicine (2009). *Status Report of Kampo Medicine Education and Doctors in Japan*.

[14] Shin HK, Bae SH (2005). A study on implication by comparing current status of educational systems between Korea and China in connection with traditional medicine of each country. *Korean Journal of Oriental Medicine*.

[15] Joe KH (2008). *Discovering Japanese Traditional Medicine*.

[16] The Uppsala Monitoring Centre Pharmacovigilance (WHO programme members). http://www.who-umc.org/DynPage.aspx?id=100653&mn1=7347&mn2=7252&mn3=7322&mn4=7442.

[17] Forum for the Harmonization of Herbal Medicines (FHH) Nature of the FHH. http://www.fhhm.net/about_us/organization.asp.

[18] Smith CA, Collins CT, Crowther CA, Levett KM (2011). Acupuncture or acupressure for pain management in labour. *Cochrane Database of Systematic Reviews (Online)*.

[19] Lee MS, Choi TY, Shin BC, Kim JI, Nam SS (2010). Cupping for hypertension: a systematic review. *Clinical and Experimental Hypertension*.

[20] Special Committee for Evidence-Based Medicine Evidence reports of Kampo treatment 2010: 345 randomized controlled trials.

[21] Laing C, Hamour S, Sheaff M, Miller R, Woolfson R (2006). Chinese herbal uropathy and nephropathy. *The Lancet*.

[22] National Center for Complementary and Alternative Medicine (NCCAM) http://nccam.nih.gov/health/whatiscam.

